# Effect of Surface Dispersion of Fe Nanoparticles on the Room-Temperature Flash Sintering Behavior of 3YSZ

**DOI:** 10.3390/ma16041544

**Published:** 2023-02-13

**Authors:** Angxuan Wu, Yuchen Zhu, Chen Xu, Nianping Yan, Xuetong Zhao, Xilin Wang, Zhidong Jia

**Affiliations:** 1 Engineering Laboratory of Power Equipment Reliability in Complicated Coastal Environments, Tsinghua Shenzhen International Graduate School, Tsinghua University, Shenzhen 518055, China; 2 Institute of Materials, China Academy of Engineering Physics, Mianyang 621907, China; 3 State Grid Jiangxi Electric Power Research Institute, Nanchang 330096, China; 4 School of Electrical Engineering, Chongqing University, Chongqing 400044, China; 5 State Key Laboratory of Power System Operation and Control, Tsinghua University, Beijing 100084, China

**Keywords:** flash sintering, room temperature, YSZ, metal powders, arc

## Abstract

Arc floating in surface flashover can be controlled by reducing the interfacial charge-transfer resistance of ceramics. However, thus far, only a few studies have been conducted on methods of treating ceramic surfaces directly to reduce the interfacial charge-transfer resistance. Herein, we explore the flash sintering behavior of a ceramic surface (3 mol% yttria-stabilized zirconia (3YSZ)) onto which loose metal (iron) powder was spread prior to flash sintering at room temperature (25 °C). The iron powder acts as a conductive phase that accelerates the start of flash sintering while also doping the ceramic phase during the sintering process. Notably, the iron powder substantially reduces the transition time from the arc stage to the flash stage from 13.50 to 8.22 s. The surface temperature (~1600 °C) of the ceramic substrate is sufficiently high to melt the iron powder. The molten metal then reacts with the ceramic surface, causing iron ions to substitute Zr^4+^ ions and promoting rapid densification. The YSZ grains in the metal-infiltrated area grow exceptionally fast. The results demonstrate that spreading metal powder onto a ceramic surface prior to flash sintering can enable the metal to enter the ceramic pores, which will be of significance in developing and enhancing ceramic–metal powder processing techniques.

## 1. Introduction

Flash sintering is a novel sintering method in which an electric field is applied to ceramic compacts to achieve rapid densification at low furnace temperatures [1]. It is different from spark plasma sintering (SPS) or other field-assisted sintering technology (FAST) in that the furnace temperature is lower (usually lower than 1000 °C), the sintering time is shorter (a few seconds), and the electric current flows through the ceramic to generate Joule heat [2,3]. As an energy-saving and cost-efficient sintering method [4,5,6,7], it requires no external heating and can be conducted at room temperature. Surface flashover can activate the room-temperature flash sintering of ceramics such as ZnO [8] and yttria-stabilized zirconia (YSZ) [9]. However, this method requires an extremely high onset voltage, higher than 3 kV/cm. Under atmospheric pressure, the arc is prone to float for a long time, usually more than 10 s, resulting in the failure of flash sintering. Arc floating can be controlled by reducing the onset flashover voltage, such as by reducing the interfacial charge-transfer resistance of the ceramic [10].

There are several ways to reduce the surface resistance, such as by using low atmospheric pressure or an atmosphere with low free electron adsorption capacity [11]; by water assistance, which has been shown to facilitate flash sintering in a reductive atmosphere (Ar + 5 mol% H_2_) at approximately 23 °C [12]; and by using conductive components, such as reduced graphene oxide and carbon nanotubes [13,14]. Owing to the addition of graphite, the FS of Sm_2_O_3_-doped CeO_2_ ceramics can be triggered at 25 °C [15]. However, there are few reports on methods of treating ceramic surfaces directly to reduce the interfacial charge-transfer resistance.

Flash joining is an attractive technique for integrating metals and ceramics [16]. YSZ ceramics are the earliest used material in flash sintering in its various industrial applications. Kiniger et al. [17] reported how flash can influence the surface properties of ceramics that interact with metal particles. He described the flash behavior of a zirconia substrate on which gold microparticles were deposited and the wetting behavior of the gold particles in the flash state. Liang et al. [18] found that the wettability of molten Cu on a 3 mol% YSZ (3YSZ) surface is significantly improved by applying a pulsed current at 1373 K. The adsorption and enrichment of oxygen could reduce the solid–liquid and liquid–vacuum interfacial energies, and hence improve the wettability. It should be noted that placing metal particles on the dielectric surface reduces the interfacial resistance of the ceramic.

In the previous study [9], we successfully achieved room-temperature flash sintering of YSZ, in order to explore the possibility of room-temperature flash sintering as a novel and easy metal–ceramic processing technology that is different from metal–ceramic interactions when metals serve as electrodes to drive currents through the ceramic. In this study, nanometer-scale Fe powder was spread on the upper surface of a 3YSZ substrate prior to flash sintering. Before the flash was ignited, the Fe phase remained in the form of a powder. During the arc maintenance stage, the powder melted owing to the high temperature of the ceramic substrate, which inhibited arc floating and significantly reduced the arc duration by improving the surface conductivity of the YSZ substrate. In addition, Fe ions entered the YSZ lattice, leading to a Fe-rich phase at the grain boundaries of YSZ. Furthermore, the YSZ grains coarsened significantly during metal infiltration.

## 2. Materials and Methods

**Figure 1:**
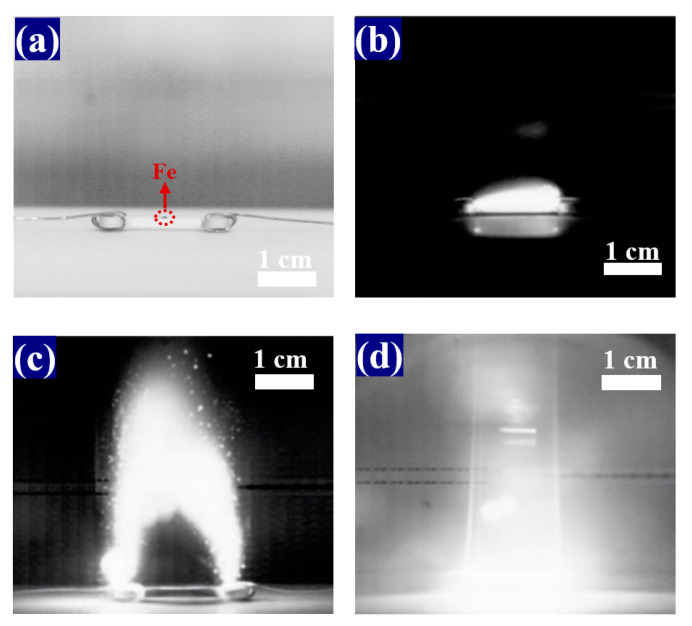
(**a**) Side view of a 3YSZ sample sprinkled with Fe powder before the flash test; (**b**–**d**) snapshots recorded using a high-speed camera during flash sintering: (**b**) 0 s, (**c**) 2.96 s, and (**d**) 8.22 s after the onset of flashover at a current density of 110.38 mA/mm^2^.

The 3YSZ powder (>99%) with average particle size of ~30 nm was purchased from Nanjing Mission New Materials Company. After mixing with 5 wt% binder (10 wt% PVA in DI water), the 3YSZ substrate was prepared by uniaxially pressing YSZ powder under 270 MPa pressure to form dogbone-shaped samples with a gauge length of 14.5 mm, width of 3.3 mm, and thickness of 1.7 mm. The samples were heated at 400 °C for 2 h to remove the binder, followed by presintering at 1100 °C for 2 h to enhance their strength. The morphological properties of the green bodies were shown in the study by [6]. The ends of the dogbone samples were coated with silver to form electrodes, which were then wound with platinum wire and connected to an alternating current (AC) power supply (YDTW 100/50, Xinyuan Electric Co., Ltd., Nanjing, China). The AC frequency, power capacity, output voltage range, and rated output current of the power supply were 50 Hz, 100 kW, 0.7–50 kV, and 2 A, respectively. The samples were then placed on an alumina board for flash sintering experiments. Iron powder (99.9%, ~200 nm, Aladdin Biochemical Technology Co., Ltd., Shanghai, China) was spread in the central region of the upper surface of the sample. The coverage of iron powder was 3.3 mm × 3 mm. Fe powder was not evenly distributed in the experiment. The amount of added Fe nanoparticles was 5 mg for each pretreated 3YSZ sample, which means the exact mass of distributed Fe nanoparticles per unit area was 50.5 mgFe/cm^2^. Photographs of a sample sprinkled with Fe powder before the flash test is shown in Figure 1a.

The flash sintering process of the samples was recorded using a high-speed camera (Phantom V2012). The temperature of the sample surface was measured using a thermal infrared camera (FLIR SC 660). The morphologies of the samples were examined using the scanning electron microscope (SEM, SU8010). The elemental composition of the samples was then characterized through energy-dispersive X-ray spectrometry (EDXS). Moreover, the crystalline structures were determined using an X-ray diffractometer (Bruker D8 Advance) with Cu Kα radiation.

When the power supply whose initial output was ~0.9 kV was turned on, flash sintering occurred as reported previously [9]. The output voltage was increased manually until the current surges, indicating the onset of flash sintering. The average voltage rising rate was controlled at <0.4 kV/s. After the flash started, the current was maintained for 30 s, then the output of the supply was reduced to a minimum and turned off.

**Figure 2:**
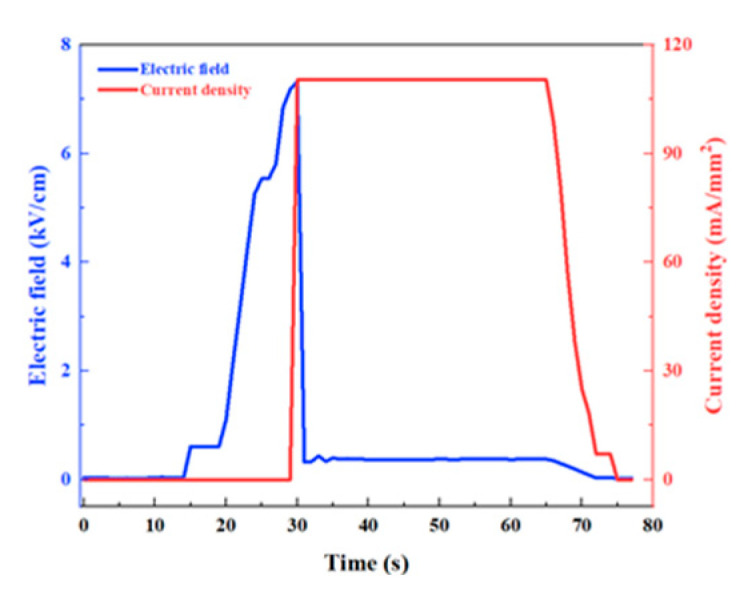
Electric field and current density as a function of time for the YSZ sample during flash sintering.

The snapshots of YSZ samples during the flash sintering are recorded in Figure 1b–d. The exposure time of these images was <100 μm to capture the electric arc that emitted extremely strong light. The room-temperature flash sintering process included three stages, namely surface flashover (Figure 1b), arc maintenance (Figure 1c), and arc settlement (Figure 1d). The electric field and current density profiles during the flash sintering process are shown in Figure 2. When the flash was ignited, the arc appeared, floated, and was extinguished periodically at 100 Hz. At the same time, the current in the circuit surged from nearly zero to 620 mA. Figure 1b presents the moment when the current surged, as shown in Figure 2. The arc completely disappeared 8.22 s after the flashover began, as shown in Figure 1d. The entire sintering process was completed within 30 s of flash ignition. For comparison, the arc duration during the flash sintering of YSZ samples without Fe powder was 13.50 s under the same experimental conditions. Thus, the presence of Fe powder on the substrate surface greatly decreased the arc maintenance time from 13.50 to 8.22 s.

**Figure 3:**
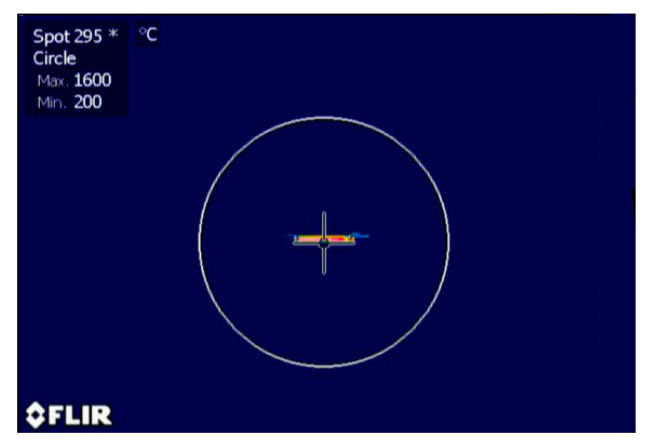
Thermal image of 3YSZ sample during the flash stage.

In the arc maintenance stage, most of the current flowed through the arc instead of through the sample. During arcing, the actual arc temperature was far beyond the range of the infrared camera, and the arc emitted strong white light, so the surface temperature could not be accurately measured. When it entered the settling stage from the arc maintenance stage, owing to the thermal effect of the arc, the surface temperature of the substrate increased to above 1600 °C, as measured by a thermal infrared camera (Figure 3). This is above the melting point of Fe (1538 °C). Consequently, it can be speculated that the metal powder begins to melt and infiltrate the upper surface of the 3YSZ substrate during arcing, which would improve the surface conductivity of the sample and thus promote the local flash sintering of the surface and accelerate the settlement of the arc.

## 3. Results and Discussion

**Figure 4:**
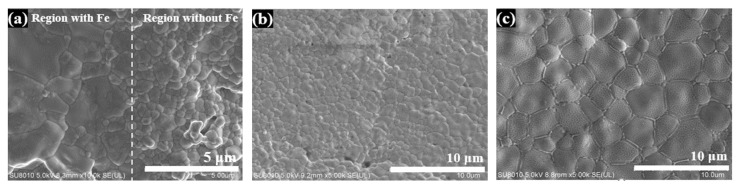

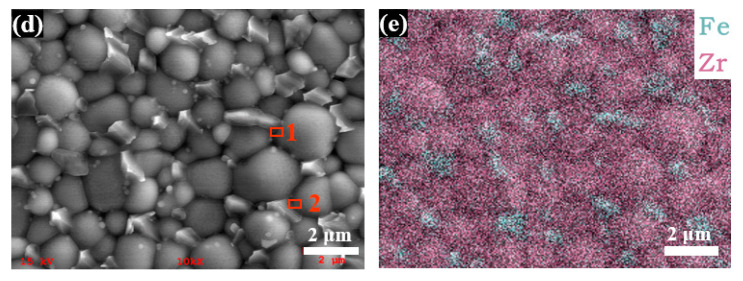
SEM images of flash-sintered 3YSZ samples with Fe nanoparticles (**a**) on the boundary between regions with and without Fe, in regions (**b**) without Fe and (**c**,**d**) with Fe; (**e**) EDS mapping image of Zr and Fe for the area in (**d**). Region 1 in (**d**) is the grain boundary region and Region 2 in (**d**) is the grain interior region.

**Figure 5:**
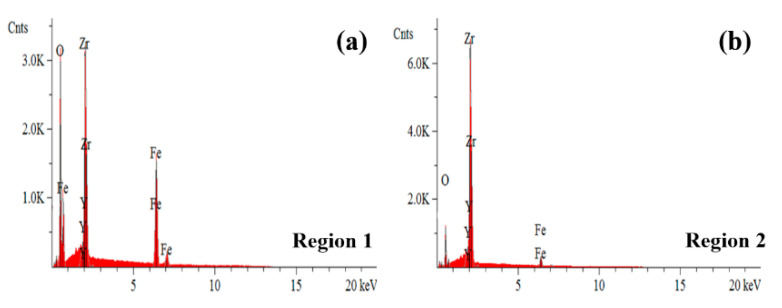
EDS patterns of flash-sintered 3YSZ samples in (**a**) grain boundary region (region 1 in Figure 4d) and (**b**) grain interior (region 2 in Figure 4d).

SEM images of the flash-sintered 3YSZ samples with Fe powder are shown in Figure 4a–d. The boundary between regions with and without Fe is indicated in Figure 4a. The regions of the surface that were not covered with Fe powder (Figure 4b) had a grain size of 1.04 ± 0.50 μm, which is smaller than that of the regions with Fe powder (4.21 ± 1.70 μm; Figure 4a). Furthermore, in the regions with Fe powder, the grain boundaries exhibited some impurities with completely different microstructures (Figure 4c), which were absent in the regions without Fe (Figure 4b). Energy-dispersive X-ray spectroscopy (EDS) analysis in Figure 4e shows that Fe was segregated at the grain boundaries and partially incorporated into the YSZ grains. EDS patterns of the grain boundary region and grain interior are shown in Figure 5, and the elemental compositions are listed in Table 1. In the grain boundary region (region 1 in Figure 4d), the Fe content was 26.13 at%. It is speculated that owing to the short flash sintering time or limited solubility of Fe in 3YSZ, a large amount of Fe could not dissolve in the grains of 3YSZ and formed Fe-rich impurities at the 3YSZ grain boundaries. In the grain interior (region 2 in Figure 4d), the Fe content was 8.86 at%, which indicates that a small amount of Zr was replaced by Fe during flash sintering. In fact, the element content in Table 1 was obtained through the peak area analysis of EDS patterns in Figure 5. The results of excessive Fe content here could not be used as accurate analysis results, but as a comparison of relative reference values.

**Figure 6:**
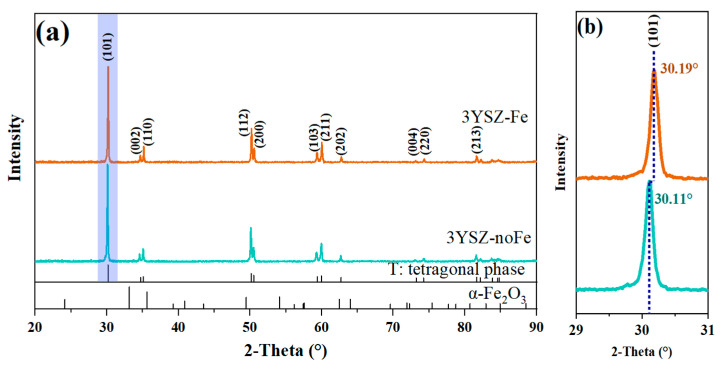
X-ray diffraction patterns of (**a**) 3YSZ samples with Fe (3YSZ-Fe) and without Fe (3YSZ-noFe) and (**b**) magnified view of the (101) peak showing the peak shift upon Fe incorporation.

Figure 6a shows XRD patterns of the flash-sintered YSZ samples in regions with and without Fe. Both regions comprised only the tetragonal YSZ phase and not the monoclinic phase, with no secondary α-Fe_2_O_3_ phase. No obvious peak was observed between the two peaks of the tetragonal phase, indicating very low content of the cubic phase. Thus, it is reasonable to state that the 3YSZ samples with a pure tetragonal phase are obtained after flash sintering, and a negligible monoclinic or cubic phase is involved. However, the peaks of the tetragonal phase were displaced in the surface region with Fe. As shown in Figure 6b, the (101) peak shifted by 0.08°. This peak shift occurs owing to the formation of a tetragonal solid solution of Zr_1−*x*_Fe*_x_*O_2−*x*/2_. The influence of Fe doping on flash sintering is discussed as below.

The radius of Fe^3+^ ions (0.55–0.78 Å) is much smaller than that of Zr^4+^ (0.84 Å), Y^3+^ (1.019 Å), and O^2−^ (1.38 Å) ions [19], indicating that Fe^3+^ doping can distort the 3YSZ crystal structure. The lattice parameters were affected by Fe^3+^ doping, with the *c*/*a* ratio of the Fe-doped region (1.437) being greater than that of the tetragonal phase (1.431) and pure 3YSZ region (1.432). According to Guo et al. [20], Fe^3+^ ions can occupy both substitutional and interstitial sites in 3YSZ. The substitution of Fe^3+^ (Equation (1)) results in the formation of oxygen vacancies ($V_{O}^{\cdot \cdot }$) to preserve the electron neutrality of the zirconia lattice [21], whereas Fe^3+^ interstitial doping (Equation (2)) generates Fe^3+^ interstitials (${Fe}_{i}^{\cdot \cdot \cdot }$) instead of oxygen vacancies [22].

(1) $${Fe}_{2}O_{3}\overset{ZrO_{2}}{\to }2{Fe\prime }_{Zr}+V_{O}^{\cdot \cdot }+3O_{O}^{x}$$

(2) $${2Fe}_{2}O_{3}\overset{ZrO_{2}}{\to }3{Fe\prime }_{Zr}+{Fe}_{i}^{\cdot \cdot \cdot }+6O_{O}^{x}$$

The substitution of Zr^4+^ with Fe^3+^ decreases the volume of the unit cell, while Fe^3+^ interstitials increase the volume. For flash-sintered 3YSZ, it seems that Fe^3+^ substitution was predominant in the doped samples, because the unit-cell volume decreased by 0.308%. It has been reported that the increased Zr^4+^ jump frequency and decreased activation energy leads to an increase in the diffusion coefficient of Zr^4+^, which results in the rapid densification of Fe_2_O_3_-doped 3YSZ/8YSZ [19]. Similarly, during room-temperature flash sintering, the substitution of Zr with Fe tends to increase the defect concentration in YSZ, accelerate grain boundary migration, and accelerate grain growth.

The above discussion is mainly based on the influence of Fe_2_O_3_-doping on zirconia. According to the research [20], different precursors and calcination sequences of the Fe_2_O_3_-Y_2_O_3_-ZrO_2_ solids can yield distinct doping sites going with different electronic valences (substitution vs. interstitial, and Fe^3+^ vs. Fe^2+^). It is discussed in [22] that iron enters the ZrO2 lattice mainly as Fe(II), allocated in roughly equal amounts in two different sites:  each of these sites is quite disordered. The sites can be identified with the regular Zr site at 0,0,0 and the normally empty site at 1/2, 1/2, 1/2. This gives rise, therefore, to roughly equal amounts of substitutional defects and interstitial defects. The pertinent quasichemical equilibrium for Fe doping can therefore be written as follows (Equation (3)).

(3) $$2FeO⇌{Fe\prime \prime }_{Zr}+{Fe}_{i}^{\cdot \cdot \cdot }+2O_{O}^{x}$$

According to [22], for the interstitial, the shorter Fe−Zr distance is only 2.64 Å. This result, coupled to the quite low value of the Debye−Waller factor relative to this distance, suggests the presence of a direct Fe−Zr metal-to-metal bond.

**Figure 7:**
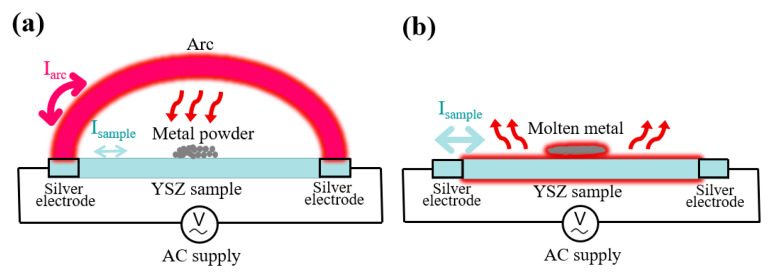
Schematic of (**a**) arc stage and (**b**) steady stage.

Figure 7 shows diagrams of the arc stage and steady stage during the flash sintering process. Before flash ignition, Fe is present as a powder. After the periodic AC arc appears, the arc current (*I*_arc_) is in competition with the current through the YSZ sample (*I*_sample_). Most of the current flows through the arc, which heats the sample surface at a rate of above 10^3^ °C/s. The surface conductivity of the sample decreases dramatically. Concurrently, the metal powder on the surface begins to melt, as shown in Figure 7a, which further improves the surface conductivity. Therefore, *I*_sample_ increases, with a corresponding decrease in *I*_arc_, thus reducing the arc maintenance time. The molten Fe on the YSZ substrate (Figure 7b) promotes densification and grain growth during flash sintering. Furthermore, the substitution of Zr by Fe generates defects including oxygen vacancies and Fe^3+^, Fe^2+^interstitials in the YSZ grains. The Zr^4+^ jump frequency increases and the activation energy decreases, both of which increase the diffusion coefficient of Zr^4+^.

## 4. Conclusions

In summary, spreading Fe metal powder on the 3YSZ surface facilitates flash sintering and accelerates the settling of the arc. Fe^3+^ replaces Zr^4+^ in the lattice, thereby promoting densification and grain growth. This work is of considerable significance for the guidance of metal–ceramic processing technologies. The flash-sintered ceramic is a high-temperature substrate, causing the metal powder to melt and diffuse into the pores of the ceramic without the need for physical pressure to force the metal into the ceramic pores. The flash-induced spreading of metal could also be combined with three-dimensional printing technologies to regulate the distribution of elements and grain growth on ceramic surfaces to fabricate novel functional gradient materials.

## Figures and Tables

**Table 1:** Elemental composition of flash-sintered 3YSZ sample in region with Fe.

Region	Element	Intensity (c/s)	Composition (at%)	Composition (wt%)
Grain boundary (region 1 in Figure 4d)	O	1038.35	56.34	22.79
	Fe	770.30	26.13	36.91
	Y	109.21	1.80	4.04
	Zr	988.31	15.72	36.26
Grain interior (region 2 in Figure 4d)	O	298.12	36.97	9.83
	Fe	175.22	8.86	8.21
	Y	174.35	4.00	5.92
	Zr	2224.74	50.17	76.03

## Data Availability

The data is clearly presented in graph form in this study. The detailed data are available on request from the corresponding author.
